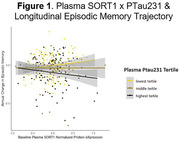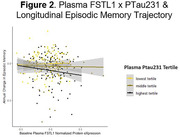# Discovery plasma proteomics analyses identify proteins that interact with plasma ptau231 levels to predict longitudinal episodic memory trajectory over a 9‐year follow‐up period

**DOI:** 10.1002/alz.093156

**Published:** 2025-01-09

**Authors:** Hailey A. Kresge, Kahraman Tanriverdi, Corey J Bolton, Antonina Risitano, Yunyi Sun, Dandan Liu, Kimberly R. Pechman, Katherine A. Gifford, Ravi Shah, Jane Freedman, Timothy J. Hohman, Angela L. Jefferson

**Affiliations:** ^1^ Vanderbilt University Medical Center, Nashville, TN USA; ^2^ Department of Neurology, Vanderbilt University Medical Center, Nashville, TN USA

## Abstract

**Background:**

There has been great progress towards identifying plasma biomarkers for Alzheimer’s disease (AD), though few studies have investigated interactions between proteomic measures and AD pathology. We aimed to identify plasma proteins predictive of episodic memory decline, an early clinical sign of AD, and assess whether such associations were altered by the presence of AD pathology.

**Method:**

Vanderbilt Memory and Aging Project participants (n=350, 73±7 years, 41% female) without dementia at baseline underwent blood draw and serial neuropsychological assessments over 9‐year follow‐up (mean=6.1 years). Proteins were quantified using Olink® Explore 3072. Linear mixed‐effects regressions related protein levels to longitudinal episodic memory composite scores, including interactions with follow‐up time as the terms of interest. Models adjusted for age, sex, race/ethnicity, education, baseline cognitive status, and apolipoprotein E‐ε4 status. Follow‐up analyses included plasma phosphorylated tau 231 (ptau231) as a covariate, tested proteins x ptau231 interactions, and stratified by ptau231 tertile.

**Result:**

Higher levels of 15 proteins (GFAP, NEFL, KLK4, SPON1, GH1, CXCL1, OGN, EPHA2, EDA2R, SHBG, CKAP4, DSG2, CD300LG, CLEC5A, NOTCH3) predicted faster decline in episodic memory (pFDR<0.05). With ptau231 included as a covariate, associations between 5 of the proteins (SHBG, CKAP4, DSG2, CD300LG, NOTCH3) and memory attenuated (pFDR>0.05). 38 proteins interacted with ptau231 to predict memory trajectory (pFDR<0.05). Among participants with high ptau23, 24 proteins (LGALS3, FSTL1, SORT1, CHAD, ADAM23, SERPINA3, COL5A1, SYT1, PTPRZ1, CD34, DPP6, POLR2F, PLAUR, SPAG1, PDE4D, PTK7, CSPG4, LRTM2, MEGF10, MYOC, REG4, SPON1, WIF1, EDDM3B) were associated with memory trajectory. Higher protein levels predicted faster decline, except for EDDM3B, for which lower levels predicted faster decline (p<0.05, pFDR>0.05). Among participants with low ptau231, lower levels of 8 proteins (FSTL1, SORT1, IL6R, PTPRM, AHNAK, AMIGO2, IL1RAP, ROBO1) predicted faster decline in memory. Two proteins (FSTL1, SORT1) were associated with memory trajectory in both low and high ptau231 groups, exhibiting opposite directions of effects in the setting of low versus high ptau231 (see Figures).

**Conclusion:**

Results demonstrate associations between plasma proteomic measures and longitudinal memory trajectory differ by level of AD pathology. To maximize clinical utility of potential biomarkers discovered in plasma proteomic analyses, future studies should assess interactions with AD core biomarkers.